# Defensive Medicine: A Case and Review of Its Status and Possible Solutions

**DOI:** 10.5811/cpcem.2019.9.43975

**Published:** 2019-10-21

**Authors:** Eric D. Katz

**Affiliations:** University of Arizona College of Medicine-Phoenix, Maricopa Integrated Health Systems, Department of Emergency Medicine, Phoenix, Arizona

## Abstract

Malpractice liability systems exist, in part, to provide compensation for medical malpractice, corrective justice for those injured by it, and to incentivize quality care by punishing substandard care. Defensive medicine is loosely defined as practice based primarily on the fear of litigation rather than on expected patient outcomes. It is largely motivated by a physician’s belief that the malpractice system is unfair, slow, and ineffective; these perceptions make malpractice concerns one of the largest physician stressors. A physician’s perception of malpractice rarely correlates with the stringency of their state’s tort system, overestimates their own risk, and overestimates the cost of defensive practices. While estimates are difficult to make, defensive medicine likely only accounts for 2.8% of total healthcare expenses. The phrase “tort reform” has been frequently used to suggest fixes to the malpractice system and to defensive practices. Safe harbors, clinical practice guidelines, comparative fault reform, reducing plaintiff attorney fees, and apology laws have each been evaluated as potential remedies to defensive practice, although most are unproven and all must be deployed in a state-by-state approach.

## INTRODUCTION

In 2004 a physician saw a patient with back pain and a leg abscess. The patient was a likeable guy, morbidly obese but dieting, and stable. He had a normal neurologic exam and a leg wound that appeared to be cellulitis in and around a venous stasis ulcer. The rate of methicillin resistance was still low in 2004 and he was treated and discharged with cephalexin and ibuprofen. When the physician was served a year later, he learned that he had missed an epidural abscess that paralyzed the patient and led to his demise nine months later. Six years later, the same physician saw a patient with atraumatic, nonspecific, thoracic spine pain, a normal neurologic exam, and a cellulitic area on the leg. He had no risk factors for perivertebral infection.

The case reminded the physician of his prior lawsuit, and while he normally would not have ordered magnetic resonance imaging (MRI) of the back, in this case he ordered the test as a result of the previous missed diagnosis. This physician was practicing defensive medicine. The test was being ordered almost entirely out of fear of litigation. There was little concern it would show an epidural. But the MRI turned out to be positive. The patient received antibiotics and surgery and did quite well. Malpractice attorneys like to say they save more lives than physicians. While physicians might strongly disagree with the statement, the lawyer who represented the first patient certainly helped the second.

While defensive medicine can lead to more cautious care, the physician is by definition less motivated by medical outcome than by legal risk. Most physicians have a strong opinion that defensive medicine results in high financial costs and unnecessary testing, and that there is an easy fix through tort reform. The literature paints a more nuanced story, with controversy surrounding the prevalence of defensive practice, the dollars spent on it, and whether or not tort reform could reduce the frequency of defensive practice. In the cases above, the defensive practice led to an unexpected but emergent finding, which can confuse the analysis even further.

Some physicians, depending on their specialty, report that they alter their practice out of fear of a lawsuit.^1^ Despite this prevalence, quantifying the cost of defensive medicine is difficult and proposing solutions for it is even harder. This paper explores the definition of defensive medicine, why it exists, its prevalence, and the costs associated with its practice. The author then reviews potential reforms that might reduce the practice of defensive medicine and discusses the limits of inferences that can be drawn from the limited data available.

## DISCUSSION

### Definitions

There are multiple definitions of defensive medicine. Kapp et. al. describes it as, “Clinical practice that is driven by the physician’s perception of legal self-interest… rather than by concern about expectation of patient benefit.”^2^ This definition paints the practice as binary and masks the complexity of medical decision-making. In many cases, concern for the patient overlaps with a physician’s personal concerns, and this overlap is not reflected in Kapp’s definition. The disbanded U.S. Congress Office of Technology Assessment defined defensive medicine this way: “when doctors order tests, procedures, or visits, or avoid certain high-risk patients or procedures, primarily (but not solely) because of concern about malpractice liability.”^3^ Both of the definitions place blame for the practice on the physician’s self-protection outweighing the patient’s needs. The latter definition allows for a gray zone where multiple factors can influence a decision simultaneously.

Defensive medicine manifests as two types of risk-avoiding behavior. Assurance behavior involves providing additional testing, hospitalization, or consultation to minimize the perceived risk to the provider.^1^ An example would be the patient described in the introduction. Avoidance behavior involves providers declining to offer complicated tests or treat potentially litigious patients in order to reduce the perceived malpractice risk.^1^ An example might be an emergency physician not wanting to ask about elder abuse when facing an assertive family member.

### Social Benefits and Physician Perception of the Liability System

All medical liability systems exist in part to provide compensation for medical malpractice, “corrective justice” for those injured (such as psychological closure), and the incentive to provide safe, quality patient care.^1^,^3^ However, as a deterrent to the unsafe practice of medicine, the tort system has been shown to be ineffective.^4^ There are those who believe defensive medicine helps encourage physicians to be more diligent,^2^ which was the case with the epidural abscess case; but this effect remains anecdotal and unquantified.

While malpractice liability has social benefit, physicians see another side of it. Physicians see the malpractice system as slow, ineffective, and biased against them.^1^,^2^,^5^,^6^ Malpractice suits are considered one of the largest physician stressors.^7^ In general, physicians’ fears of the malpractice system are only loosely correlated with the actual stringency of their state’s malpractice tort system,^6^,^8^ and often are in excess of risks.^9^ For example, in the five states with the highest malpractice risk, 68% of physicians reported engaging in defensive medicine. Yet in the five states with the lowest malpractice risk, the number only decreases to 64%.^8^ In addition, while there is a belief that a physician’s own malpractice experience shapes his or her degree of malpractice avoidance, studies do not confirm this tendency.^1^,^5^ In the epidural abscess case, previous malpractice experience altered future care.

### Quality of Evidence and Confounding Variables

While opinions on the presence and magnitude of defensive medicine are profound, there is little evidence to support those opinions. The majority of studies of defensive medicine in Medline and Westlaw (57%) were based on physician surveys, with only 9% based on primary statistical analysis and 7% on literature reviews (mostly of survey studies).^2^ Many studies are based on a single specialty or specific disease (such as heart attacks, spinal disc disease, etc.). The presence of author bias is palpable on all sides of the issue.

Medical decision-making is a complex process that incorporates defensive medicine with other influencers. Those influencers include quality care, financial incentive, patient satisfaction, self-image, professional reputation, and the desire to avoid conflict. Isolating any one variable is exceptionally difficult, and most surveys cannot single out malpractice concerns except through hypothetical simulation. Many use graded scales of perceived malpractice risk to try to simulate situations in which defensive medicine can be identified. Others will attempt to quantify the respondents’ malpractice-avoidance and correlate that with costs.^6^,^10^

### Quantifying the Cost of Defensive Medicine

While almost every physician survey shows defensive medicine to be ubiquitous,^1^–^3^,^5^,^6^,^8^,^10^,^11^ the total cost and percentage of orders affected is unclear. On in-patient medical services, it is estimated that 2.9% of costs are purely defensive, and another 10.1% are somewhat defensive.^10^ One inpatient-based study showed that spending more reduced malpractice risk. Providers with higher hospitalization cost (mean $39,379) had a 0.3% risk of claims per year, while those with lower costs (mean $19,725) had a 1.5% risk of claims per year.^1^ In this case, assurance behavior was effective at reducing malpractice risk.

The total cost of defensive practice ranges from $46 billion to $300 billion, although most estimates are between $50–65 billion.^5^ This is less than 3% of total healthcare costs. Those in the extreme will claim up to 25% of healthcare costs are generated by defensive medicine,^12^ although that number is a high-end estimate of total healthcare waste, of which defensive practice is only one element. Mello et al. provided one of the most detailed assessments and fully recognized the multiple assumptions made. They concluded that 2.8% of healthcare costs were defensive in nature (in 2008), which equated to $55 billion.^3^

### Effect of Tort Reform

It has been proposed (usually by physicians) that significant tort reform could decrease defensive medical practice and thereby decrease medical costs. If true, this would allow physicians to be more comfortable making decisions without undue psychological pressure to mitigate malpractice risks. Unfortunately, in addition to overestimating the actual potential savings, physicians likely underestimate the obstacles to this approach.

#### Caps on non-economic damages

There is conflicting evidence as to whether tort reform changes practices. For example, an oft-quoted study from 1996 showed that caps on non-economic damages (“pain and suffering”) for Medicare patients reduced hospital costs in patients with myocardial infarcts and ischemic heart disease.^13^ Later studies using the same methodology and same patient type did not confirm this finding.^14^ Mello and Kachalia reviewed several caps on non-economic damages with a conclusion that reducing non-economic damage ceilings had an indeterminate effect on healthcare spending, although there were reductions in spending in some subgroups of spending (e.g., a slower rate in the growth of malpractice premiums, possible reduction in defensive practice, and compensation awards).^15^ Bioethicist Ezekiel Emanuel criticizes malpractice caps as not reducing healthcare spending and increasing the risk that patients injured by negligence might not be fully compensated,^8^ thereby undermining some of the beneficial social effect of malpractice.

#### Capping attorney fees

In 2017, an analysis of state malpractice reforms and their effects on malpractice showed the only reform that decreased physician spending on insurance was capping attorney contingency fees.^14^ As attorney fees are generally 35–40% of awards (after costs),^3^ and the attorney often pays the costs out of pocket, decreasing the contingency fee reduces attorney profits, while keeping their costs the same. This de-incentivizes attorneys and ameliorates physician anxiety by reducing the total number of cases, particularly those with lower potential judgments. In theory, plaintiffs would retain the ability to achieve “corrective justice,” although it would be harder to find representation, and the financial structure might change. Should the plaintiff win, he or she would keep more of the judgment than under the current fee system. If one assumes that the cause of defensive medicine is an overabundance of frivolous lawsuits, it is a reasonable approach.

#### Comparative fault versus contributory negligence

Comparative fault reforms, when enacted, significantly increased malpractice cost.^14^ Comparative fault is a tort law concept in which providers are held liable for the proportional percentage of damages based on their contribution to the outcome. 14 The provider may be held liable, even if the patient’s actions contributed to an untoward outcome. Comparative fault reforms usually replaced contributory negligence rules, in which if a patient was at 1% (or greater) fault for an injury, the patient could not obtain and damages.^14^ It is not surprising that the effect was increasing cost of coverage.

The changes seen in Yu’s study were in cost of insurance.^14^ It is important to note that the cost of insurance is based on factors other than the amount of defensive medical spending. The rates are in large part set based on risk assessment by malpractice carriers. In this sense, they are a proxy for measuring the degree of physician risk of a malpractice case.

#### Safe harbors for evidence-based guidelines

One proposal that may have merit is the creation of “safe harbors” for evidence-based guidelines.^5^,^8^ In these harbors, using clinical practice guidelines (CPG) developed under rigorous quality standards would protect the physician from legal judgment. Some national organizations, such as the American Board of Internal Medicine with its *Choosing Wisely* campaign, have led the push for this approach. They seek to improve quality while reducing defensive practice. There is survey evidence that doctors do not trust CPGs to legally protect them,^2^ but there currently are no safe harbor laws in place to test this theory. The guidelines can be used by defendants and expert witnesses to show a standard of care, although the protective effect of CPGs has not been measured.

#### Apology laws

Apology laws have been enacted in several states to allow physicians to apologize for errors or poor outcomes without the apology being admissible in court. In theory, apologies reduce patient anger and maintain trust, thereby reducing claims. Meaningful data on their efficacy is lacking, although many have called for further exploration.^8^

#### Malpractice-specific courts

Specialized courts for healthcare have also been proposed, although they have not been adopted in any state. Healthcare courts could provide a layer of consistency to diminish physician concerns about unfair treatment by a jury of lay people. While the jury makeup might not change, the governance of the courts would theoretically be more reproducible.^8^ When all considerations are included, the Congressional Budget Office analysis concluded that the total percentage of healthcare expenses that could be saved through tort reform would be a paltry 0.3%.^11^ For this reason, nontraditional approaches, such as communication and resolution, judge-directed negotiation, and administrative compensation systems, are being explored by the Agency for Healthcare Research and Quality.^16^

### Medical and Legal Case Outcome

The epidural abscess patient passed away nine months after his emergency department visit. He was paralyzed below the diaphragm for most of that time. It is unclear whether his course would have been any different had the physician made the diagnosis. While there are always areas for improvement, the physician treated him the same way as almost every other doctor would have, and only the most defensive (or brilliant) diagnostician would have ordered an MRI on him. The case was settled for $400,000. The physician did not try to force it to trial.

## CONCLUSION

While malpractice serves a social benefit, it also creates stress for physicians and an increase in the practice of defensive medicine. The result is approximately 2.8% of medical expenses being spent to avoid litigation, rather than benefit patients. While this is a small percentage, it is a large dollar amount. Tort reform has limited potential to impact the practice.
